# Recent Developments in Transition-Metal Catalyzed Direct C–H Alkenylation, Alkylation, and Alkynylation of Azoles

**DOI:** 10.3390/molecules25214970

**Published:** 2020-10-27

**Authors:** Su Chen, Prabhat Ranjan, Leonid G. Voskressensky, Erik V. Van der Eycken, Upendra K. Sharma

**Affiliations:** 1 Laboratory for Organic & Microwave-Assisted Chemistry (LOMAC), Department of Chemistry, University of Leuven (KU Leuven), Celestijnenlaan 200F, B-3001 Leuven, Belgium; su.chen@student.kuleuven.be (S.C.); prabhat.ranjan@kuleuven.be (P.R.); 2 Organic Chemistry Department, Science Faculty, Peoples’ Friendship University of Russia (RUDN University), Miklukho-Maklaya street 6, RU-117198 Moscow, Russia; lvoskressensky@sci.pfu.edu.ru

**Keywords:** azoles, C–H functionalization, transition metal-catalysis, C–H activation, alkenylation, alkylation, alkynylation

## Abstract

The transition metal-catalyzed C–H bond functionalization of azoles has emerged as one of the most important strategies to decorate these biologically important scaffolds. Despite significant progress in the C–H functionalization of various heteroarenes, the regioselective alkylation and alkenylation of azoles are still arduous transformations in many cases. This review covers recent advances in the direct C–H alkenylation, alkylation and alkynylation of azoles utilizing transition metal-catalysis. Moreover, the limitations of different strategies, chemoselectivity and regioselectivity issues will be discussed in this review.

## 1. Introduction

Azoles are important heterocyclic scaffolds because of their wide application in pharmaceuticals, natural products, and functional materials [1,2,3]. After the first report of the antifungal activity of azoles in 1944 by Woolley, their functionalization and synthesis started attracting the attention of researchers [4]. The functionalization of azoles has been well elaborated for decades by classical metalation with strong bases such as *n*-BuLi [5]. However, these classical methods suffer from general limitations, such as low chemo- and/or regio- selectivity, low atom economy, sophisticated reaction conditions, and more importantly, poor reactivity in the case of electron-deficient azoles [6]. Moreover, the cost of waste disposal and the need to handle reactive bases make these methods unsuitable for industrial scale-up. As a consequence, over the last decade, continuous demand for these scaffolds in the pharmaceutical industry and academia has resulted in several new approaches, mainly based on C–H functionalization.

Direct transition-metal catalyzed C–H functionalization is an effective tool for streamlining azole-based biologically important frameworks [7,8,9,10,11]. However, the presence of heteroatoms in an azole framework renders the overall C–H activation more challenging and substrate-specific, owing to their ligation tendency with transition-metal catalysts. Besides the heteroatom issue, the inherent electrophilic nature of azoles favors either a nucleophilic attack via ring opening, or a direct attack in the presence of metal catalysts. Furthermore, the alkylation of azoles with alkyl halides bearing a *β*-hydrogen is challenging due to the possibility of *β*-hydride elimination and/or hydrodehalogenation in the presence of transition metal catalysts. Similarly, the alkenylation of azoles with alkenyl halides is problematic due to the tendency of homocoupling of alkenyl halides, as well as the low stability of moisture-sensitive iodides and triflates. In this context, researchers have proposed several strategies to overcome these challenges, such as: (1) the use of directing groups to control regioselectivity, (2) the development of moisture-sensitive coupling partners such as phosphates and carboxylic acids, and (3) modulation of the innate reactivity of azoles via electronic and steric modifications.

Nevertheless, this field needs close inspection for the development of stable and reactive electrophilic partners with improved catalytic systems. Recently, Evano and Theunissen [12] highlighted the importance and challenges associated with the alkylation of various heteroarenes. Ackermann and coworkers reviewed the alkenylation and alkylation of azoles, especially via 3*d* transition metals [13]. Many reviews have been published on C–H functionalization/activation strategies [14,15,16]; to the best of our knowledge, none of them is truly focused on the direct C–H bond functionalization of azoles. The continuously increasing demand for new azole-based antifungal agents still requires precise control of regioselectivity and cost-efficient catalytic systems. Therefore, throughout this review, we highlight the role of transition metals in the direct C–H alkylation, alkenylation, and alkynylation chemistry of azoles. We substantiate the importance of these processes and outline common mechanistic patterns, focusing primarily on the formation of new C–C bonds. 

## 2. Alkenylation

The alkenylation of azoles has attracted much attention due to their widespread use in polymers and materials chemistry [17]. There are four main synthetic approaches for transition-metal-mediated alkenylation of azoles: (1) C–H/C–X alkenylation (X = halogen or triflate) of azoles with alkenyl halides catalyzed by palladium or other transition metals; (2) C–H/C–H alkenylation (oxidative Mizoroki–Heck reaction [18]) of azoles with simple alkenes catalyzed by palladium and rhodium catalysts; (3) C–H addition of azoles to alkynes catalyzed by transition metal catalysts (i.e., Rh, Pd, and Ni); and (4) decarbonylative C–H alkenylation. For clarity, we will discuss all the recent pioneering developments in each category with some important mechanisms, as well as shortcomings.

### 2.1. C–H/C–X Cross-Coupling

**Scheme 1:**
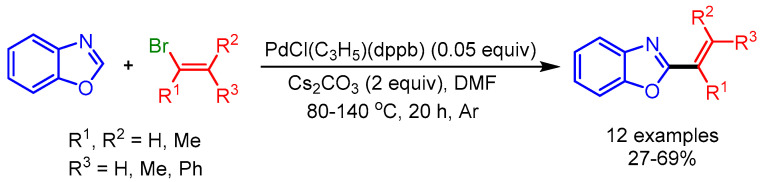
Direct Pd-catalyzed alkenylation of azoles with bromoalkenes.

Straightforward C–H/C–X cross-coupling is a classical and one of the most studied methods for the alkenylation of azoles, due to the wide availability of diverse coupling partners (i.e., halides and triflates). Moreover, the preparation of an organometallic coupling partner can be avoided with this approach. In 2008, Doucet and coworkers reported an early example of the alkenylation of azoles with C–H/C–X cross-coupling (Scheme 1) [19]. The authors employed *α*- or *ß*-substituted alkenyl bromides for palladium-catalyzed coupling of electron-rich benzoxazoles, benzothiazoles, or 2-*n*-propylthiazoles to afford the desired products via C–H bond activation. They performed the reaction at high temperature (80 °C to 140 °C) and under an inert atmosphere (Ar) in the presence of PdCl(C_3_H_5_)(dppb) as the catalyst. They overcame the decomposition of heterocycles otherwise observed at high temperatures by using an excess quantity thereof. Interestingly, this process produced minimal waste; i.e., HX associated with a base, instead of a metallic salt, rendering it intriguing in terms of both atom economy and nontoxic waste.

**Scheme 2:**
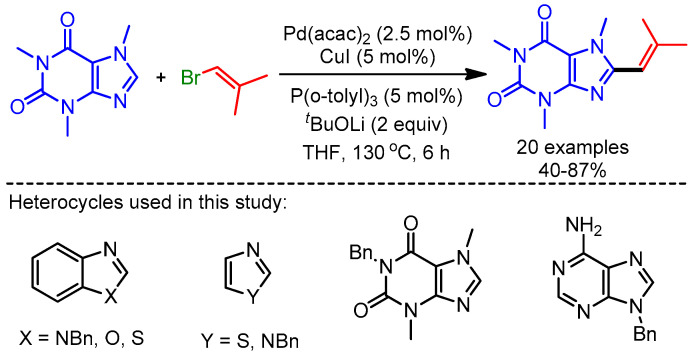
Pd/Cu-catalyzed direct alkenylation of azole heterocycles with alkenyl bromides.

Alami and coworkers employed a bimetallic Pd/Cu catalytic system for the direct alkenylation of azoles with alkenyl halides to afford the corresponding 2-vinyl-substituted azoles in moderate to good yields [20]. The reaction was not successful in the absence of CuI. Many heterocycles including benzimidazole, benzoxazole, benzothiazole, and thiazole derivatives afforded the desired alkenylated products with mono-, di-, or trisubstituted alkenyl bromides in good yields (Scheme 2). 

**Scheme 3:**
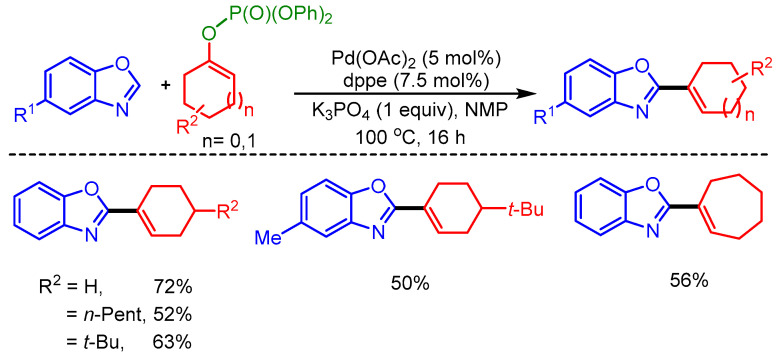
Direct alkenylation of azoles with phosphates.

In 2010, Ackermann and coworkers reported Pd-catalyzed alkenylation of benzoxazoles by using moisture-stable alkenyl phosphates as coupling partners. However, the scope of the reaction was limited to only five examples (Scheme 3) [21].

**Scheme 4:**
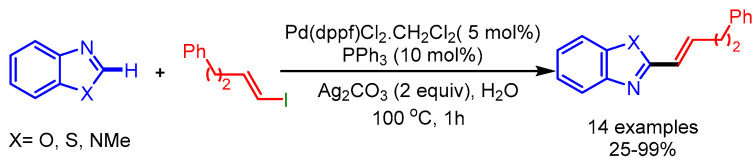
Pd-catalyzed direct functionalization of benzoxazoles with alkenyl iodides.

The microwave-assisted synthesis utilizing H_2_O as solvent is a cost-efficient and green approach in organic synthesis. In 2012, Willis and coworkers employed such an approach for the coupling of various alkenyl iodides with benzoxazoles using [Pd(dppf)Cl_2_.CH_2_Cl_2_], PPh_3_, and Ag_2_CO_3_ as the catalytic system [22] (Scheme 4). They coupled a wide range of alkenyl iodides to afford the desired products with good regioselectivity.

**Scheme 5:**
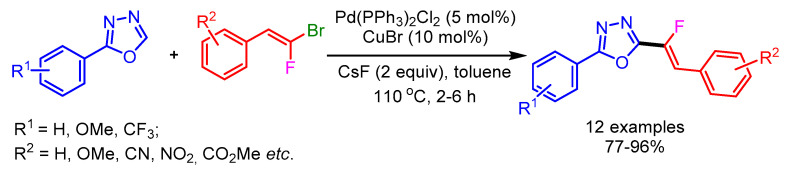
Direct C–H fluoroalkenylation of 2-phenyl-1,3,4-oxadiazole.

Owing to the importance of monofluorinated alkenes in several areas, Hoarau and coworkers developed a Pd^0^/Cu^1^ bimetallic catalytic system for stereospecific direct C–H alkenylation of various 1,3-diazoles utilizing *gem*-bromofluoroalkenes as electrophiles (Scheme 5). A range of electron-withdrawing and donating groups on *gem*-bromofluoroalkenes successfully afforded the desired products in good yields without producing the undesired alkynylated product. The authors used strong bases such as Cs_2_CO_3_ and *^t^*BuOLi for alkenylation of less-acidic 1,3-diazoles to furnish the desired monofluorinated products [23].

**Scheme 6:**
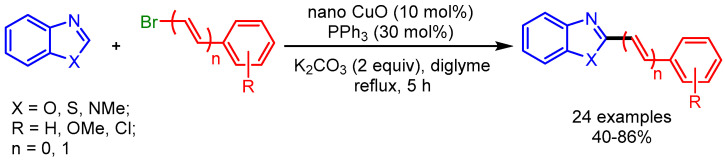
Nano CuO-catalyzed C–H functionalization of azoles with bromoalkenes.

Recently, nanoparticles have emerged as a robust catalytic system for many organic transformations [24,25,26], providing an alternative to conventional homogenous catalysis. In 2014, Wang and coworkers employed CuO (6.5 nm) nanoparticles for alkenylation of benzoxazoles, benzothiazoles, and 1-methylbenzimidazoles with alkenyl bromides (Scheme 6) [27]. The authors proposed a mechanism similar to the homogeneous catalytic system reported by Miura [28], in which PPh_3_ stabilized the surface of the nanoparticles and acted as a ligand. Moreover, the catalyst can be used several times without losing its catalytic activity.

**Scheme 7:**
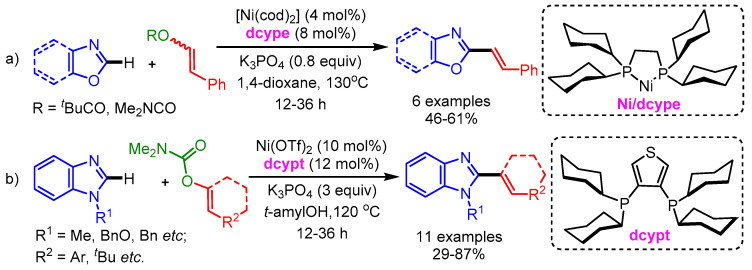
Ni-catalyzed alkenylation of azoles with (**a**) enol derivatives and (**b**) carbamate.

In 2013, Yamaguchi and Itami disclosed an efficient nickel-catalyzed C–H/C–O coupling of heteroarenes and enol derivatives [29]. A 1,2-bis(dicyclohexylphosphino)ethane (dcype) ligand was necessary for this Ni(0) catalytic system (similar ligands displayed an extremely low or no promoting effect; Scheme 7a). They employed styryl pivalate and carbamate as coupling partners with various azoles such as oxazoles and benzoxazoles with substituents at the C5 position (methyl, methoxy, and *t*-butyl) to afford the corresponding styrylated products in moderate to good yields. Moreover, the authors showed the utility of this new method by synthesizing Siphonazole B in a convergent manner as compared with previous reports. However, benzothiazoles did not react with styryl pivalates and carbamates under the optimized reaction conditions. Later in 2015, the same group improved the efficiency of this reaction by employing a Ni(II)/dcypt catalytic system to deliver the alkenyled imidazoles in high yields (Scheme 7b) [30].

**Scheme 8:**
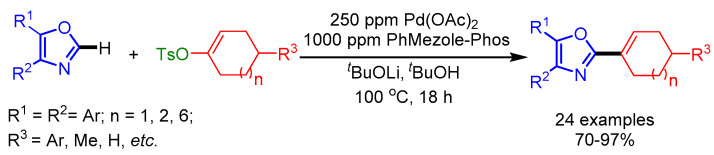
Direct C2-alkenylation of oxazoles with alkenyl tosylates.

In 2016, Kwong and coworkers disclosed a Pd/PhMezole-Phos-catalyst system for alkenylation of oxazoles by employing readily available and stable alkenyl tosylates as coupling partners [31]. They reduced the catalyst loading to 250 ppm, providing an opportunity to minimize the residual Pd-content in pharmaceutical products. Moreover, they synthesized a new class of Ir(III) complexes by using C2-alkenyloxazoles, useful for applications such as cell imaging. In addition, they carried out a gram-scale synthesis without altering the reaction conditions, affording the desired product in 85% yield (Scheme 8).

**Scheme 9:**
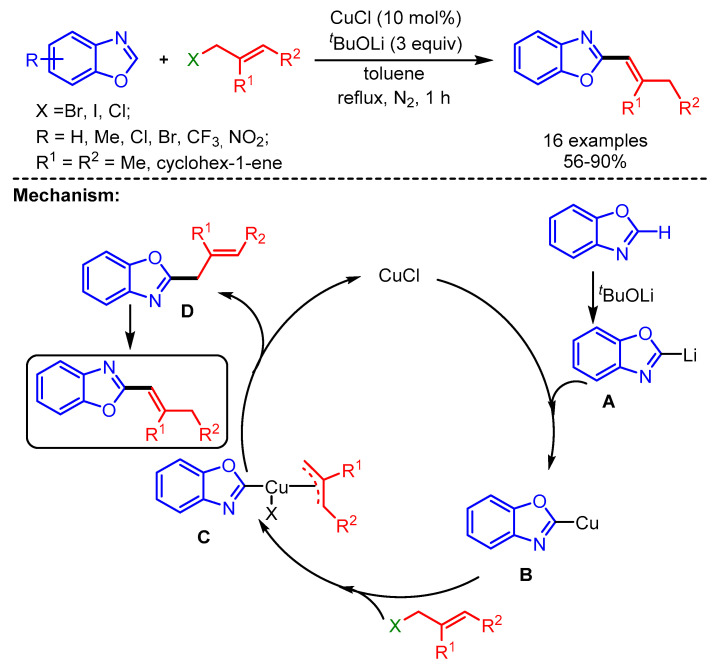
CuCl-catalyzed direct C–H alkenylation of benzoxazoles with allyl halides.

A year later, Chen and coworkers described an efficient method for C2-alkenylation of benzoxazoles by using an economical Cu-atalytic system and allyl halides as coupling partners under ligand-free conditions [32]. Interestingly, slightly modified reaction conditions produced a more conjugated 2-(buta-1,3-dienyl)benzo[*d*]oxazole product in 75% yield from 1,4-dibromobut-2-ene and benzoxazole. The reaction starts with deprotonation of the benzoxazole followed by metalation to form intermediate **B**. The in situ intermediate then undergoes oxidative insertion with the allyl halide to generate intermediate **C**, which further undergoes reductive elimination to afford the desired product (Scheme 9).

**Scheme 10:**
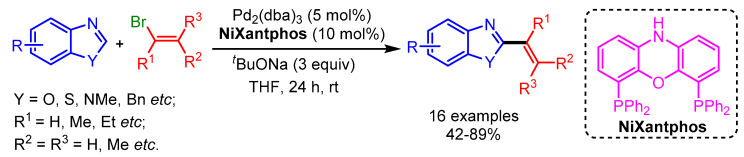
Ni-catalyzed direct alkenylation of azole derivatives with alkenyl bromides.

In 2019, Liang and coworkers followed Doucet’s Pd-catalytic approach for C2-alkenylation of a variety of azole-based heterocycles by using a more efficient Ni–xantphos catalytic system (Scheme 10) [33]. These modified reaction conditions presented a moderate scope for C2-alkenylation. However, a higher reaction temperature was needed for sterically hindered bromoalkenes.

### 2.2. C–H/C–H Cross-Coupling

From the viewpoint of sustainable and atom-economic development, C–H/C–H cross-coupling offers the most straightforward approach in alkenylation of azoles. Also, substrate pre-activation is not required.

**Scheme 11:**
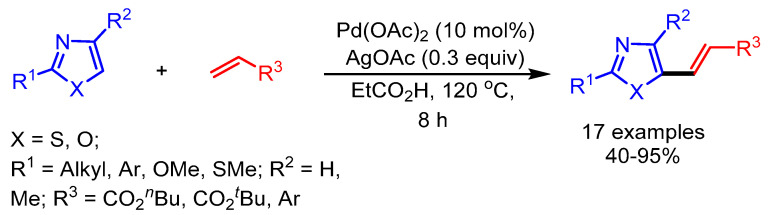
Pd-catalyzed direct oxidative alkenylation of azoles.

Transition-metal-catalyzed oxidative olefination (typically from acrylates) of heteroarenes for direct C–C bond formation via C–H bond cleavage is also known as the Fujiwara–Moritani reaction. In 2010, Miura and coworkers reported direct alkenylation of 2-substituted azoles using Pd(OAc)_2_ as catalyst and AgOAc as oxidant to deliver the corresponding 5-alkenylated azoles in moderate to good yields (Scheme 11) [34]. Internal or aliphatic alkenes were not suitable under the optimized reaction conditions and gave lower yields. They exemplified the utility of the process by synthesis of *π*-conjugated 2,5-dialkenylated thiazoles, which showed interesting solid-state fluorescence properties.

**Scheme 12:**
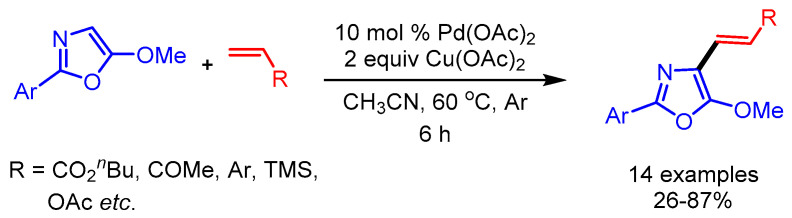
Pd-catalyzed C4-olefination of oxazoles.

In 2011, Antilla and coworkers replaced Ag with a cheaper Cu oxidant for C4-olefination of oxazoles catalyzed by Pd(II) acetate via C–H bond activation under mild reaction conditions (Scheme 12) [35]. They applied their optimized reaction conditions to a wide substrate scope including various olefins such as electron-deficient alkyl acrylates, *N*,*N*-ethylphenylacrylamide and substituted styrenes, with good functional group tolerance. Interestingly, more-challenging substrates such as vinyl trimethylsilane and 1-phenyl-1,3-diene also afforded the desired products in moderate to good yields.

**Scheme 13:**
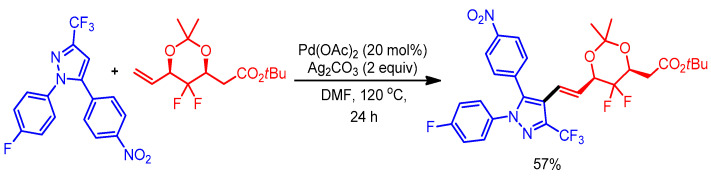
Pd-catalyzed reaction of 1,3,5-trisubstituted pyrazoles with *gem*-difluoromethylenated acetonide.

*Gem*-difluoromethylene exhibits extraordinary biological activities with potential pharmaceutical applications [36]. Owing to its importance, Wu and coworkers reported synthesis of pyrazole derivatives containing a *gem*-difluoromethylene moiety through a Pd-catalyzed direct *o*-olefination of CF_3_-substituted pyrazoles (Scheme 13) [37].

**Scheme 14:**
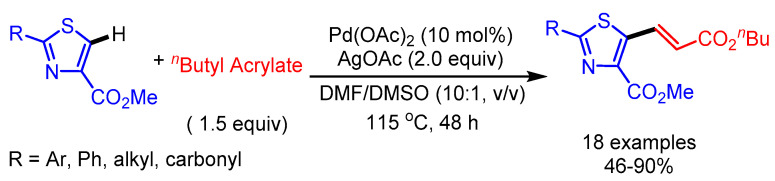
Pd(II)-catalyzed oxidative Heck coupling of thiazole-4-carboxylates.

Yao and coworkers reported a Pd(II)-catalyzed oxidative Heck coupling of thiazole-4-carboxylates to deliver the desired alkenylation products in moderate to good yields (Scheme 14) [38]. This C–H functionalization uses neither ligands nor acidic additives to accelerate the reaction. Thiazoles containing 2-alkyl and 2-carbonyl substituents mainly gave homocoupling byproducts with *n*-butyl acrylates.

**Scheme 15:**
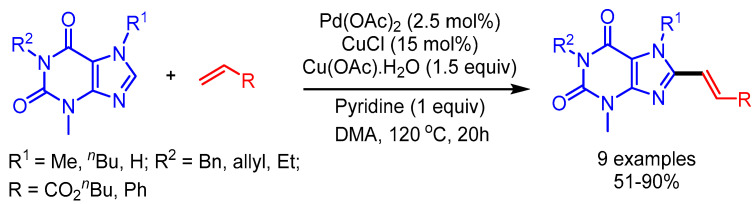
Pd/Cu-catalyzed dehydrogenative alkenylation of caffeine derivatives.

In 2012, You and coworkers reported an efficient trimetallic catalytic system using Pd(OAc)_2_/CuCl/Cu(OAc)_2_·H_2_O for a dehydrogenative Heck coupling, achieving direct alkenylation of various biologically relevant *N*-heteroarenes with alkenes [39]. They applied their reaction conditions to caffeine and xanthine derivatives, and afforded the desired products in good to moderate yields (Scheme 15). The authors highlighted the importance of this protocol by showing the fluorescence emission ability of these new scaffolds. 

**Scheme 16:**
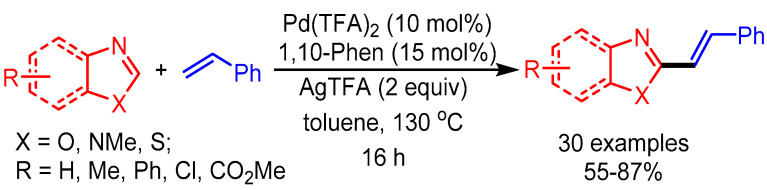
Pd-catalyzed dehydrogenative alkenylation of azole motifs.

Inspired by You’s work, in 2014 Ong and coworkers employed a Pd(TFA)_2_ and 1,10-phenanthroline catalytic system with Ag as oxidant for C-2 alkenylation of azoles (Scheme 16). The authors revealed the considerable role of C–H bond cleavage of the alkene in the rate-determining step. Moreover, they achieved direct and efficient synthesis of naturally occurring Annuloline and Siphonazole, showing a promising opportunity in natural product synthesis [40].

**Scheme 17:**
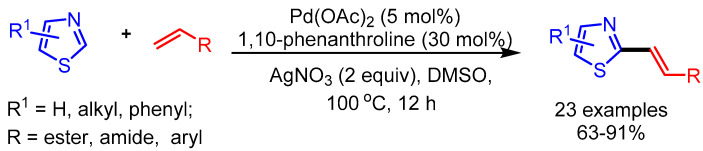
Pd-catalyzed C2-selective olefination of thiazoles.

Kuang and coworkers performed an extensive experimental study for C2-selective alkenylation of thiazoles. The overall selectivity of this process relies on the neutral reaction conditions and the ligand choice (1,10-phenanthroline). The authors presented a broad substrate scope with good functional group tolerance (Scheme 17) [41].

**Scheme 18:**
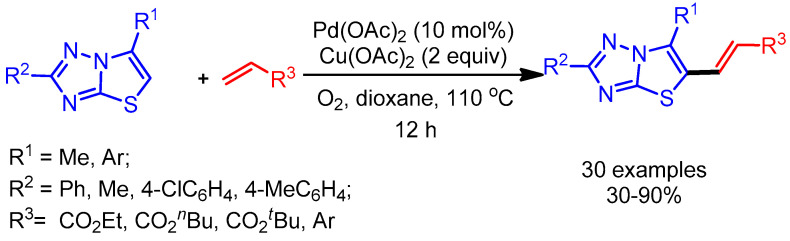
Pd-catalyzed direct alkenylation of thiazolo[3,2-b]-1,2,4-triazoles.

Thiazolo[3,2-b]-1,2,4-triazole moieties are valuable molecules that exhibit a wide range of biological activities [42]. In 2014, Wang and coworkers reported an atom-economical approach to obtain alkenylation of thiazolo[3,2-b]-1,2,4-triazoles via a palladium-catalyzed, two-fold C–H functionalization [43]. Interestingly, the synergetic effect of a metal oxidant, Cu(OAc)_2_, with oxygen plays an important role in the C–H activation step, affording the desired products in high yields (Scheme 18).

**Scheme 19:**
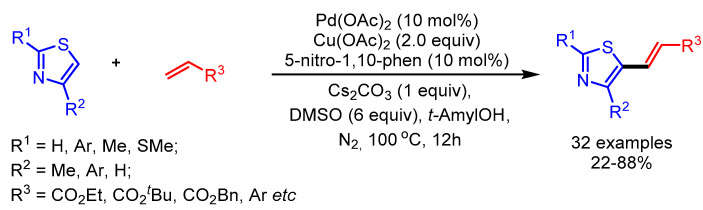
Synthesis of multifunctionalized thiazole derivatives via regioselective and programmed C–H activation.

In 2015, Shi and coworkers employed a semi-continuous approach to overcome the long-standing homocoupling problem in direct oxidative cross-coupling to functionalize thiazole derivatives (Scheme 19) [44]. Interestingly, *^t^*AmylOH and DMSO played an important role in increasing the yield of the desired products. The authors showed a broad substrate scope with good to moderate yields.

**Scheme 20:**
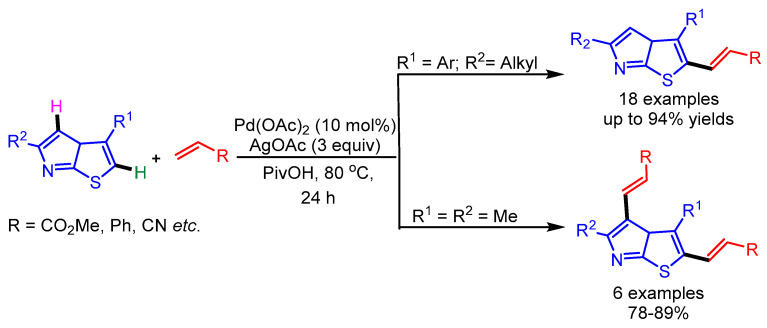
Pd-catalyzed site-selective C–H alkenylation of imidazo[2,1-b]thiazoles.

In 2016, Huang and coworkers utilized a well-established Pd-catalyzed direct C–H bond functionalization strategy for dehydrogenative alkenylation of imidazo[2,1-b]thiazole derivatives (Scheme 20) [45]. The reaction conditions were compatible with a wide range of alkenes and thiazoles.

**Scheme 21:**
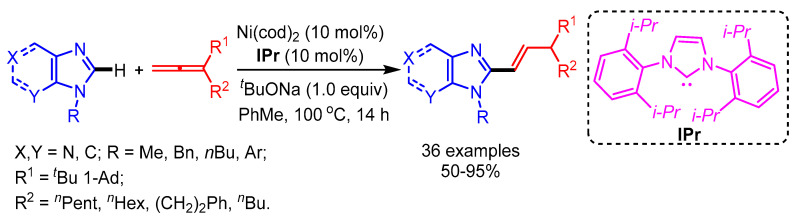
Ni-catalyzed functionalization of imidazoles with allenes.

In 2017, for the first time, Ackermann and coworkers introduced a Ni-catalyzed regioselective hydroarylation of allenes with purines and imidazoles [46]. The authors successfully applied this catalytic system for dienylation through C–H functionalization and concurrent C–O cleavage (Scheme 21). The employed base plays a key role in the isomerization step to deliver the desired products. The practical utility of this protocol was shown by the late stage-diversification of diphophodiesterase inhibitors.

**Scheme 22:**
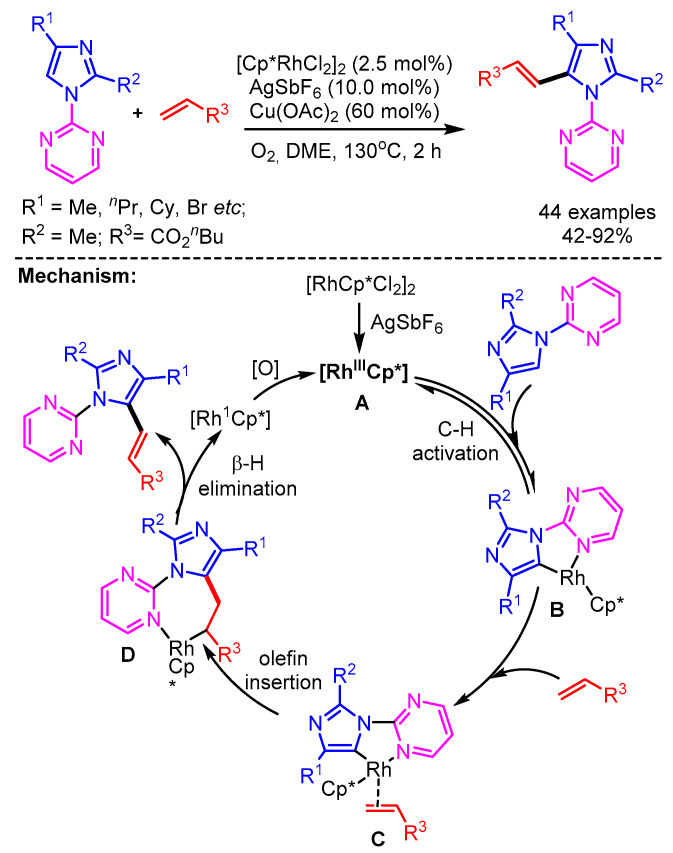
Rh (III)-catalyzed selective direct olefination of imidazoles.

In 2018, Xu and coworkers employed cationic rhodium(III) for the direct olefination of imidazoles with high regio- and stereo- selectivity [47]. Unlike the other reported methods, this catalytic system requires a directing group for the selective C-2 alkenylation. A broad range of benzimidazoles and activated olefins successfully delivered the desired products in good yields. In the proposed mechanism, the reaction starts with the Rh(III)-mediated C–H bond activation to form the cyclorhodium intermediate **B**, followed by coordination with olefin **2** to give intermediate **C**. Then a regioselective migratory insertion of the olefin delivers the complex **D**. The *β*-H elimination furnishes the desired product **3** and Rh(I). The Rh(I)-species is further oxidized by O_2_, regenerating Rh(III) to complete the catalytic cycle (Scheme 22). 

**Scheme 23:**
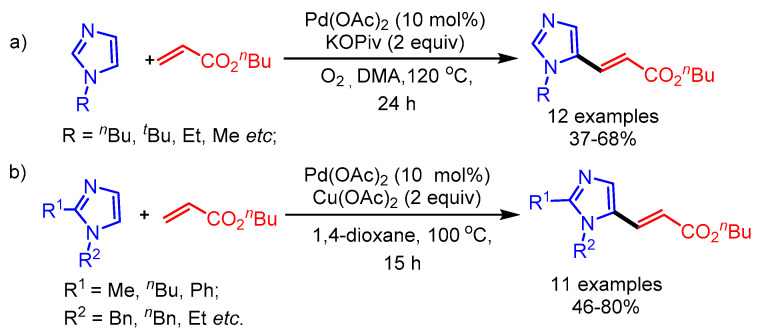
Regioselective C–H alkenylation for (**a**) C2-unsubstituted and (**b**) C2-substituted imidazoles.

Later on, Joo and coworkers reported a systematic access to functionalized (benz)imidazoles via direct C–H functionalization [48]. Molecular oxygen as an oxidant was found essential for the selective C5-alkenylation of imidazoles. In this study, the authors highlighted the distinctive role of imidazole as ligand for Pd(II) to regioselectively install alkenyl groups at the more nucleophilic C5 position of the imidazoles (Scheme 23a,b). Notably, the reaction conditions were not compatible with related 1,3-azoles, i.e., thiazoles and oxazoles. Furthermore, the installed C5-alkenylated imidazoles could be subjected to additional alkenylation, affording unsymmetrically substituted benzimidazoles.

**Scheme 24:**
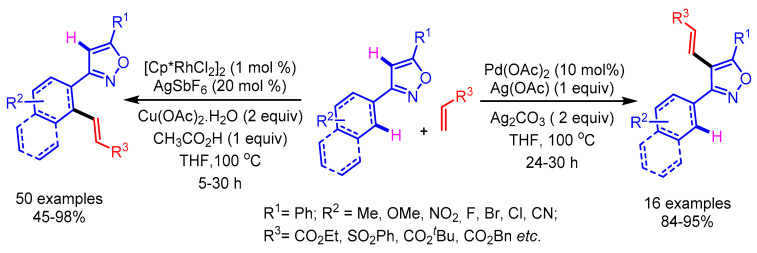
Catalyst control in positional-selective C–H alkenylation of isoxazoles.

Recently, Kumar and Kapur developed an interesting catalyst-driven selective C–H functionalization of isoxazoles at the distal and the proximal position as shown in Scheme 24 [49]. The mode of activation in the case of cationic Rh involves the strong coordination with the isoxazole nitrogen, favoring the proximal C–H activation of the arene ring. On the contrary, the Pd-catalyst prefers to undergo the electrophilic metalation at the C(4)-H of the isoxazole to furnish the desired distal C–H activation. Furthermore, the kinetic isotope studies elucidated the possibility of carbometallation or the *β*-hydride elimination to be the rate-limiting step, instead of the C–H activation step. The synthetic utility of this position-selective C–H olefination approach was demonstrated by the synthesis of densely substituted pyrroles by employing a Ru- and Cu-mediated cooperative catalysis. 

**Scheme 25:**
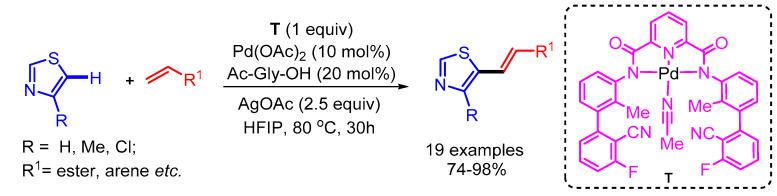
Pd-catalyzed template-directed C-5 selective olefination of thiazoles.

The regioselective alkenylation of 2,5-unsubstituted thiazole derivatives has been a challenging issue among synthetic chemists. Maiti and coworkers overcame this challenge by using a tricoordinating directing group (T) bearing a cyanide template for the selective metalation of thiazole at the C5 position followed by reductive elimination to deliver the desired products [50]. The authors presented a wide range of substrate scope with good functional group tolerance (Scheme 25).

### 2.3. C–H Addition of Azoles to Alkynes

**Scheme 26:**
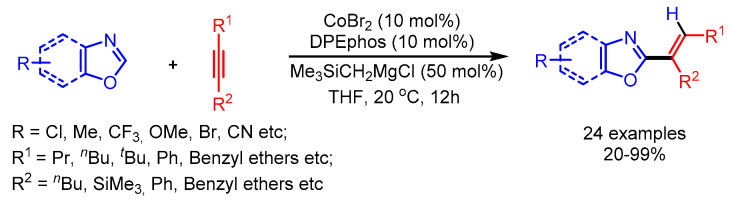
Co-catalyzed addition of benzoxazoles to alkynes.

Alkynes are potentially good substrates for alkenylation because of their wide availability and low cost. The transition metal-catalyzed direct cleavage of the C–H bond of azoles followed by insertion of alkynes appears an ideal and atom economical method for the alkenylation of azoles. This straightforward synthetic strategy was early reported by Miura [51]. Later, in 2009, Miura and coworkers reported a Ni-catalyzed C–H alkenylation of 1,3,4-oxadiazoles [52]. At the same time, Hiyama and coworkers also reported a similar approach for the alkenylation of imidazoles [53]. In 2010, Ding and Yoshikai reported a Cobased catalytic system for the addition of benzoxazoles to unactivated internal alkynes (Scheme 26) [54]. The optimized reaction conditions showed good regio- and chemo- selectivity possibly originating during the alkyne insertion step. Unfortunately, the optimized conditions were not suitable for the alkenylation of benzoxazoles with terminal alkynes. 

**Scheme 27:**
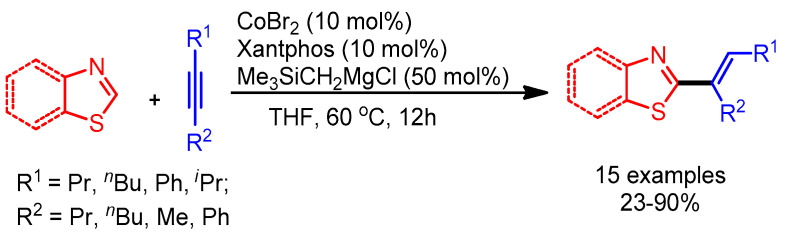
Co-catalyzed alkenylation of thiazoles with alkynes.

Later, Ding and Yoshikai observed the chemoselective alkenylation of (benzo)thiazoles in the presence of (benz)oxazoles by changing the ligand from DPEphos to Xantphos. The authors could not provide the reason for the observed selectivity, but their work opens the door for further investigations (Scheme 27) [55].

**Scheme 28:**
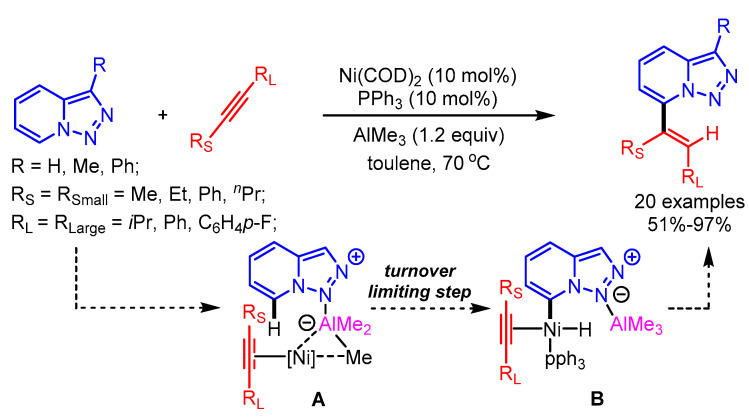
Ni-catalyzed alkenylation of triazolopyridines.

The selective alkenylation of triazolopyridines using a Ni-catalytic system was reported by Driver and coworkers. In this pioneering work, the authors highlighted the steering role of a Lewis acid (AlMe_3_) interaction for the selective oxidative addition of the C7-H bond to generate intermediate **B**, followed by alkyne insertion to obtain the desired products (Scheme 28) [56]. The alkyne insertion step was mainly curbed by the substituent steric factor on the alkyne bond, favouring the new C–C bond between the smaller alkynyl substituent and the C7 of the triazolopyridine.

**Scheme 29:**
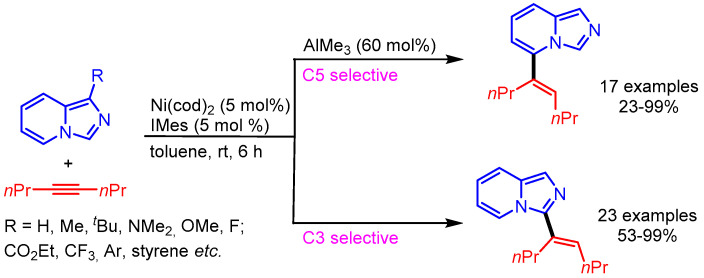
C–H alkenylation of imidazo[1,5-a]pyridine with alkynes.

Similar to this report, Ong and coworkers employed the same Ni/Al bimetallic catalytic system for the remote C–H functionalization of imidazo[1,5-a]pyridines. Interestingly, the authors were able to switch the regioselectivity of alkenylation from C5 to C3 by excluding AlMe_3_ from the catalytic system (Scheme 29) [57]. A wide scope was presented with good functional group tolerance. 

**Scheme 30:**
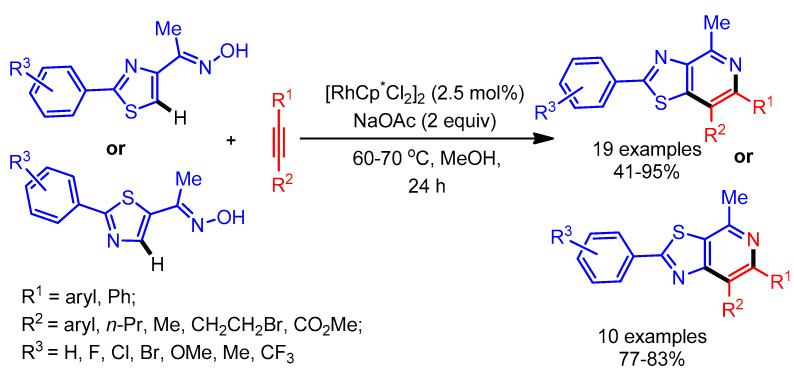
Rh(III)-catalyzed cyclization reaction of azoles with alkynes.

In 2015, Huang and coworkers exploited an oxime as directing group in the selective alkenylation of thiazoles employing a Rh-catalyst. This was followed by a tandem cyclization to deliver azole fused pyridines (Scheme 30) [58]. Despite having lower reactivity at the C-4 position, various thiazoles underwent smooth alkenylation with alkynes. In general, electron-rich alkynes gave better yields than electron deficient alkynes. Notably, this catalytic system also successfully delivered the desired products with unsymmetrical alkynes.

**Scheme 31:**
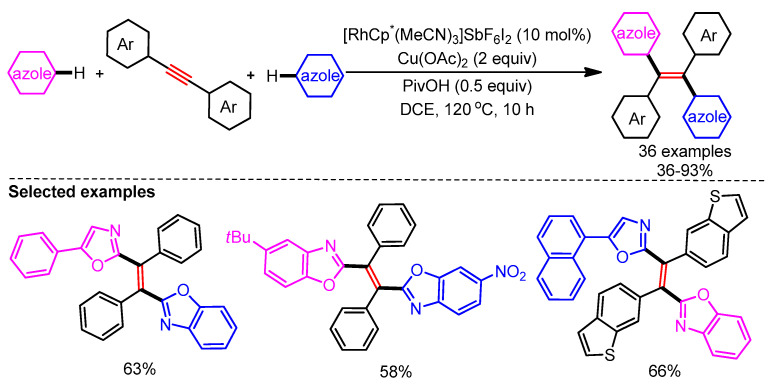
Rh/Cu-catalyzed highly trans-selective 1,2-diheteroarylation of alkynes with azoles.

In 2017, You and coworkers exploited a Rh/Cu-catalytic system for the alkenylation of azoles via a C–H addition/oxidation sequence to generate tetra(hetero)arylethylenes (Scheme 31). Interestingly, control experiments and DFT calculations revealed that the C–H bond cleavage of azoles could be facilitated by either Rh or Cu. The authors also supported the necessity of Cu(OAc)_2_ to obtain the desired product by computational studies. Tetra-arylethylene (TAE) derivatives have widely been used in OLED applications. This makes the protocol highly alluring for future developments in the area of functional materials [59]. Employing this catalytic system, a broad range of alkenylated azoles could be readily generated in good yields from alkynes.

**Scheme 32:**
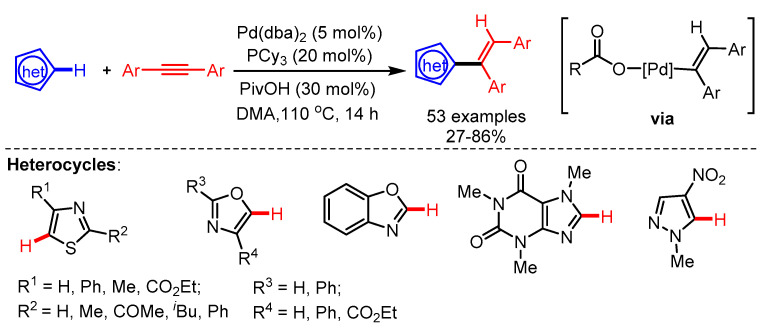
Pd-catalyzed *syn*-hydroarylation reaction of diaryl alkynes with azoles.

Another interesting approach for the alkenylation of the C5-position of thiazoles using a Pd/PCy_3_/RCO_2_H-catalytic system was disclosed by Joo and coworkers in 2019 [60]. The key intermediate of this catalytic cycle is the alkenyl Pd(II)-carboxylate complex, which enables the concerted metalation deprotonation of substituted azoles, followed by reductive elimination to deliver the corresponding products. The reaction conditions were not only compatible with substituted thiazoles but also with oxazoles, benzoxazoles, and pyrazoles (Scheme 32). The hydroarylation of azoles occured at the C2 position if the C5 position was not available. 

**Scheme 33:**
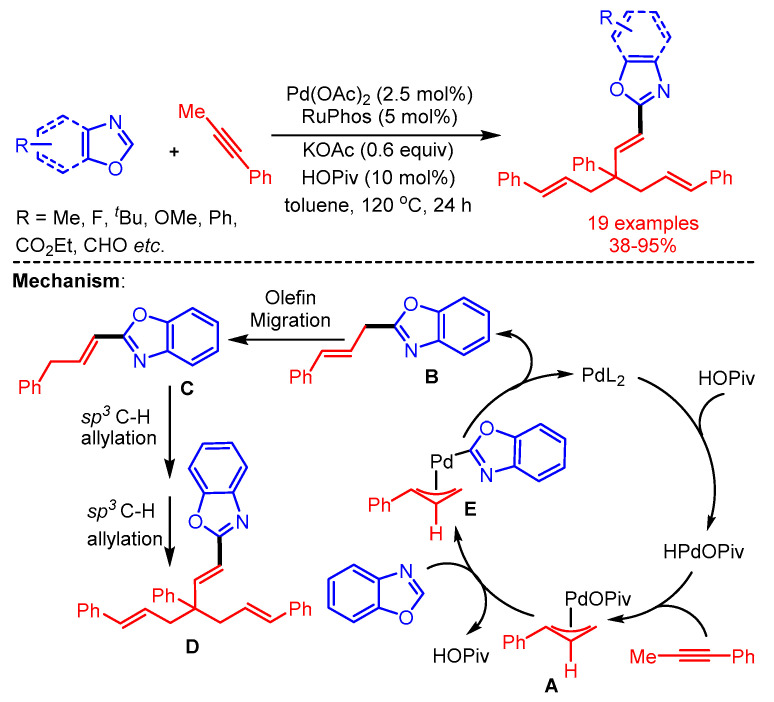
Pd-catalyzed trisallylation of benzoxazoles with an alkyne.

In 2019, Breit and coworkers introduced an interesting cascade sequence to achieve the trisallyation of benzoxazoles catalyzed by Pd(OAc)_2_ using Ruphos as ligand [61]. In the proposed mechanism, the reaction starts with the generation of a Pd-hydride species through oxidative addition of the Pd-catalyst with HOPiv. Then, the Pd-hydride species facilitates the isomerization of the alkyne to allene and affords the *π*-allylpalladium intermediate. Subsequently, intermediate **A** reacts with azoles by C–H activation to produce mono-allylated product **B**, followed by isomerization and sequential two-fold C(sp3)–H allylation with *π*-allylpalladium intermediate **A** to furnish the desired product **D** (Scheme 33).

### 2.4. Decarbonylative C–H Alkenylation

**Scheme 34:**
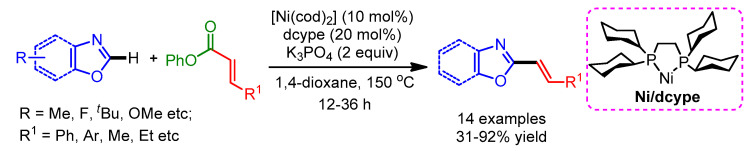
Ni-catalyzed decarbonylative C–H alkenylation of various azoles.

In recent years, decarboxylative C–H bond functionalization has drawn growing attention, since carboxylic acids are commercially available and are structurally diverse. Obviously, the use of transition-metal catalyzed vinyl carboxylic acids as olefin sources with heterocycles via direct activation of C–H bonds is an attractive and atom-economic synthetic methodology. Several transition-metal coupling methodologies for the direct decarboxylative olefination have been reported [62,63]. Decarbonylative cross-couplings for the alkenylation of azoles have remained elusive for some time. In 2013, Itami and coworkers reported an inimitable method for the alkenylation of azoles with a Ni/dcype catalytic system, using enol derivatives and *α*,*β*-unsaturated esters [29]. Moreover, this catalytic system can also efficiently enable decarbonylative alkenylation with enol derivatives of styryl pivalate, styryl carbamate and phenyl cinnamate (Scheme 34). In general, the coupling product of various azoles with carbamates delivered the corresponding products with higher yields as compared to styryl pivalates.

**Scheme 35:**
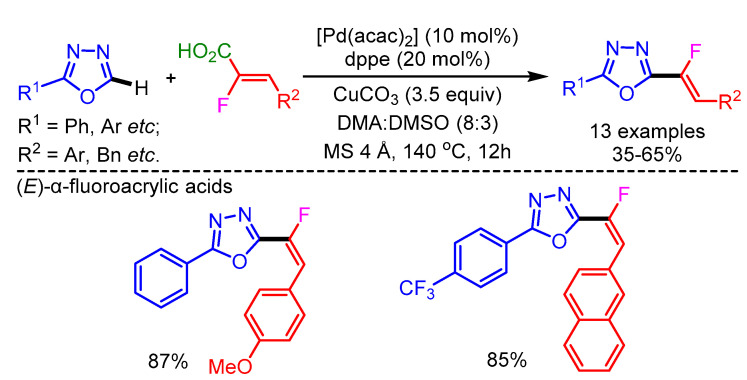
Decarboxylative/direct C–H monofluoroalkenylation with fluoroacrylic acids.

Fluorinated heterocycles have always been attractive scaffolds for pharmaceuticals and organic materials. In 2014, Hoarau and coworkers reported an efficient method for the direct C–H/C–CO_2_H cross-coupling between azoles and *α*-fluoroacrylic acids via a decarboxylative process, using a Pd/Cu-catalytic system [64]. The reaction mechanism is believed to start with a Cu-promoted decarboxylation step generating an alkenylcopper intermediate. This is followed by a transmetalation step with the Pd-azole intermediate. Finally, reductive elimination generates the desired product. The various (*Z/E*)-*α*-fluroacrylic acids successfully delivered the *Z/E*-isomeric products in good to moderate yields with good functional group tolerance (Scheme 35). 

**Scheme 36:**
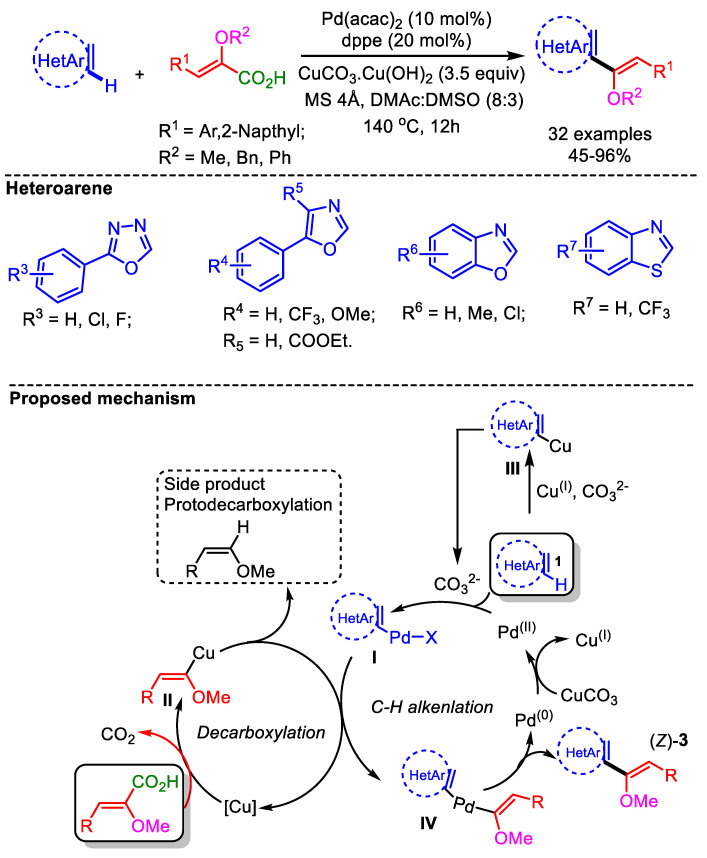
Pd-catalyzed *α*-carboxyenol ether formation with various azoles via direct decarboxylative cross-coupling and proposed mechanism.

Later in 2017, the same group developed a decarboxylative C–H alkenylation of various azoles with *α*-alkoxylated acrylic acids [65]. This decarboxylative coupling proceeded smoothly employing [Pd(acac)_2_] as catalyst along with CuCO_3_.Cu(OH)_2_. This transformation displays good *E*/*Z* stereochemistry and *α*/*β* regiochemistry. For less acidic benzothiazoles, CuI played a key role in increasing the acidity at the C2-H of the heteroarene, forming a [Cu]-heterocycle. Therefore, depending on the acidity and the nucleophilicity of the azole, there could be two possible pathways to activate the azoles and afford intermediate (**I**): (i) direct Pd-catalyzed heteroarenes (azoles) via a base-assisted concerted or nonconcerted (carbanionic-type) process, or (ii) heteroarylcopper(**III**) through a transmetalation step with Pd(OAc)_2_ (Scheme 36). Using these optimized conditions, the authors successfully reported a convenient approach to generate *α*,*β*-enolizable *α*-ketoazoles and biologically active C2−C4′ linked azoles.

## 3. Alkylation of Azoles

Friedel-Craft alkylation has been used as the first-line technique for the alkylation of azoles before direct C–H functionalization came on the scene [6]. However, Friedel-Crafts alkylation mostly encounters several difficulties such as low chemo-and/or regioselectivity, being limited to electron-rich substrates, and harsh reaction conditions [12]. C–H activation is providing an efficient tool for the direct alkylation of azoles with almost surgical precision. Great contributions by Fagnou, Miura and Ackermann in the field of azole alkylation via C–H activation have made this field wide open for further developments [66,67,68].

### 3.1. Primary C–H alkylation of Azoles

**Scheme 37:**
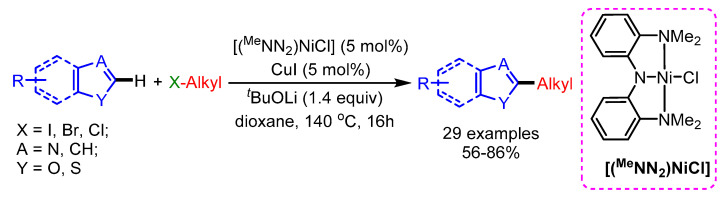
The Ni/Cu-catalyzed direct alkylation of heterocyclic C–H bonds.

Among different C–H alkylations of azoles, primary alkylation has been widely investigated using halides, pseudohalides and alkenes as coupling partners. Among the various metals used, it is no surprise that Pd adopts a key role, though other metals like Rh, Ni and Cu are catching up, and some astonishing discoveries have been made in recent years. Alkylation of heteroarenes through the direct cross-coupling with nonactivated alkyl halides containing a *β*-hydrogen atom, has been a challenging issue. In 2010, Hu and coworkers reported a novel nickel complex for the alkylation of the C–H bond of azoles with nonactivated alkyl halides containing a *β*-hydrogen atom, using CuI as a cocatalyst (Scheme 37) [69]. A wide range of functional groups branching at the *β*-position of the halides showed good reactivity, and high chemo- and regioselectivity. 

**Scheme 38:**
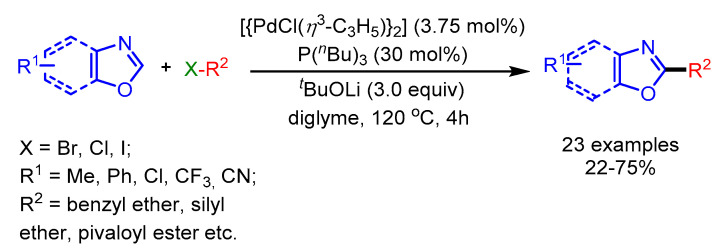
Pd-catalyzed direct C–H alkylation of benzoxazoles with various alkyl halides.

Concurrently, Miura and coworkers reported a Pd-catalyzed direct alkylation of oxazoles with nonactivated alkyl halides [70]. A variety of alkyl bromides possessing a benzyl ether, a silyl ether or a pivaloyl ester functional group, transformed into the desired products in moderate to good yields (Scheme 38). Moreover, unactivated alkyl chlorides were also well tolerated employing this catalytic system. Unfortunately, 1-iodohexane gave a much lower yield (20%) while bromocyclohexane could not provide the product due to the rapid decomposition and steric hindrance, respectively. 

**Scheme 39:**
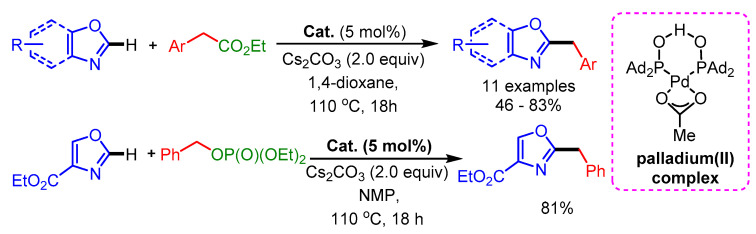
Benzylations on oxazol(in)es with a Pd- catalyst of a secondary phosphine oxide.

In 2011, Ackermann and coworkers reported the alkylation of oxazoles with benzyl chlorides and benzyl phosphate applying a Pd(II) complex [71]. The authors found that phosphinous acid Pd(II) complex significantly improved the yield in comparison with other palladium catalysts. In the presence of a prefunctionalized Pd(II) complex, a wide range of differently substituted benzyl chlorides reacted efficiently with (benz)oxazoles, providing the corresponding products in moderate to high yields (Scheme 39).

**Scheme 40:**
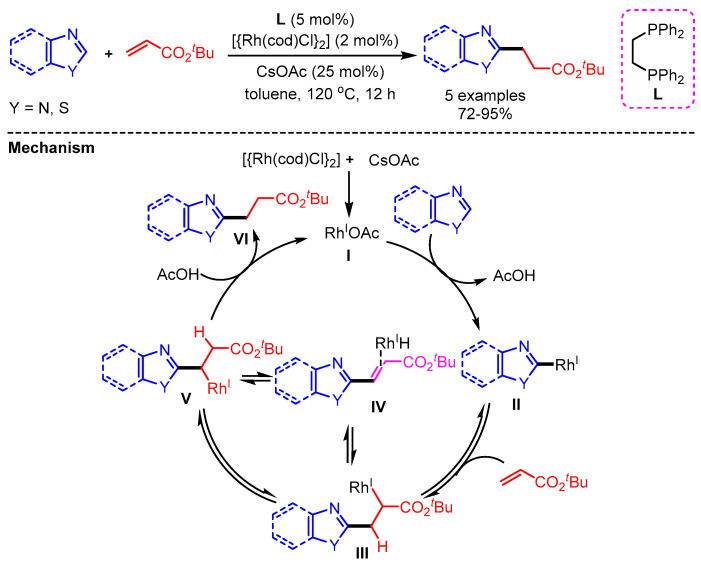
Rh- catalyzed alkylation by the addition of azoles to alkenes.

In 2012, the first example of direct addition between azoles and alkenes via C–H bond activation of heteroarenes was reported by Chang and coworkers using a [{Rh(cod)Cl}_2_] catalyst (Scheme 40) [72]. A possible reaction pathway follows (i) formation of a mono-rhodium species **I** via ligand exchange with cesium acetate, (ii) base-promoted deprotonation and metalation to generate the Rh/heteroaryl species **II**, (iii) olefin insertion, (iv) *β*-hydride elimination and re-insertion affording intermediated **IV** and **V** respectively, and final protonation to deliver products **VI** with regeneration of the Rh(I) species.

**Scheme 41:**
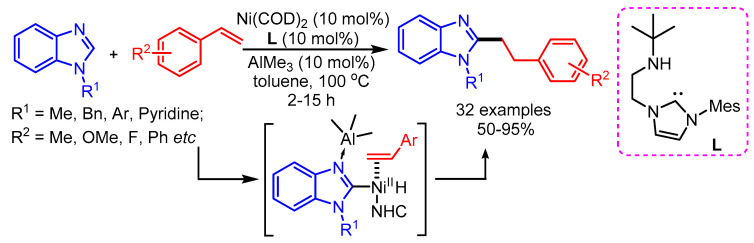
Amino-NHC mediated C–H activation of benzimidazole via Ni-Al synergistic catalysis.

Styrene can act as an efficient alkylating reagent. In 2012, the Ong group reported a Ni-Al bimetallic catalyzed alkylation of benzimidazoles with styrenes to deliver linear products [73]. The reaction was successfully performed by using Ni(COD)_2_/AlMe_3_ cooperative catalysis along with amino-NHC (**L**) as ligand (Scheme 41). Like in the previous reports, the binding of the Lewis acid (AlCl_3_) to the benzimidazole favors the linear chain product due to steric hindrance.

**Scheme 42:**
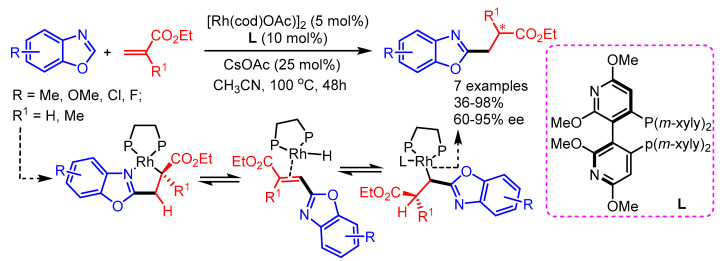
Rh(I)−bisphosphine catalyzed asymmetric, intermolecular hydroheteroarylation of *α*-substituted acrylate derivatives with benzoxazoles.

In 2015, Filloux and Rovis for the first time introduced the enantioselective alkylation of benzoxazoles with *α*-substituted acrylates using a Rh(I)/chiralphosphine catalytic system to afford 2-substitued benzoxazoles in moderate to excellent yields with good enantioselectivities [74]. Mechanistically, Rh(I)-acetate first activates the C–H bond of the benzoxazole and migratory insertion follows, providing a Rh-enolate complex. Then, *β*-H elimination and hydrorhodation deliver the heterobenzyl-Rh intermediate. The authors proposed the crucial role of bulky chiral ligands to steer enantioselectivity in the desired products by discouraging undesired ligation of heterocycles or by attenuating coordination-promoted product epimerization (Scheme 42).

**Scheme 43:**
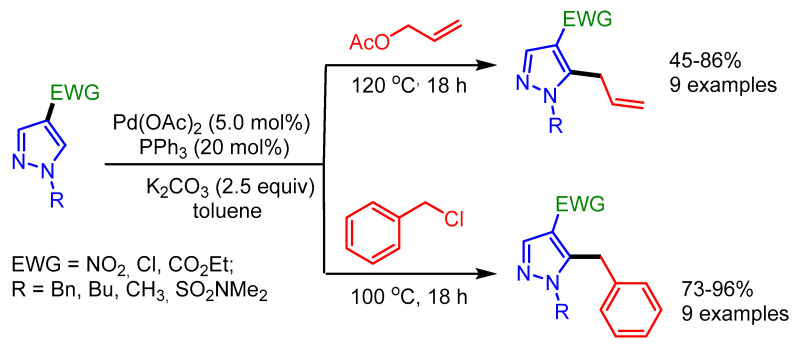
Pd-catalyzed C–H allylation and benzylation of pyrazoles.

In the same year, Joo and coworkers explored allylation and benzylation reactions of pyrazoles by installing an electron-withdrawing group such as a nitro, a chloro, or an ester group at the C4 position. Such substitution decreases the Lewis basicity of nitrogen atom and renders the C–H bond more acidic, thus enabling the Pd-catalyzed regioselective C–H functionalization (Scheme 43) [75].

**Scheme 44:**
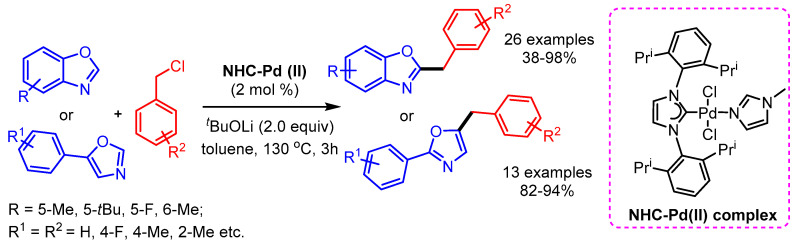
NHC-Pd(II) complex-catalyzed benzylation of (benz)oxazoles with benzyl chlorides.

Concurrently, Shao and coworkers reported a phosphine free NHC-Pd(II) complex-catalyzed direct C–H bond benzylation of (benz)oxazoles with benzyl chlorides [76]. The authors presented a broad substrate scope with good functional group tolerance (Scheme 44).

**Scheme 45:**
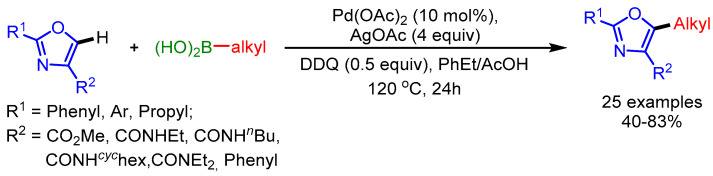
DDQ-promoted direct C5-alkylation of oxazoles with alkylboronic acids.

In 2017, Li and coworkers described a C5-alkylation of oxazoles with alkylboronic acids using a Pd(OAc)_2_/AgOAc catalytic system with DDQ as oxidant (Scheme 45) [77]. DDQ facilitates the regeneration of Pd(II) in the catalytic cycle.

**Scheme 46:**
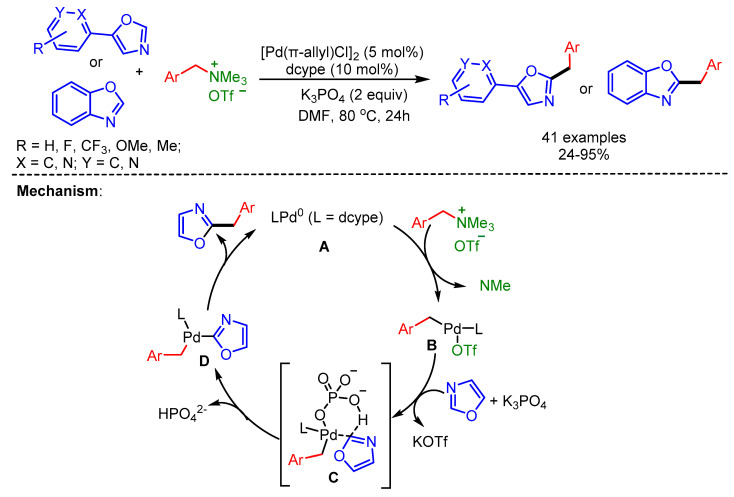
Pd-catalyzed C–H benzylation of (benz)oxazoles with benzylic quaternary ammonium triflates and a plausible mechanism.

In 2018, Pan and Wang explored the use of quaternary ammonium triflates as a coupling partner for the benzylation of (benz)oxazoles in the presence of a Pd/dcype/K_3_PO_4_-catalytic system [78]. Mechanistically, in situ generated Pd(0) undergoes oxidative addition with ArCH_2_NMe_3_^+^OTf^−^ to form the intermediate **B**. Subsequent ligand exchange and C–H activation forms the intermediate **D** via intermediate **C**. Finally, reductive elimination delivers the desired product (Scheme 46).

**Scheme 47:**
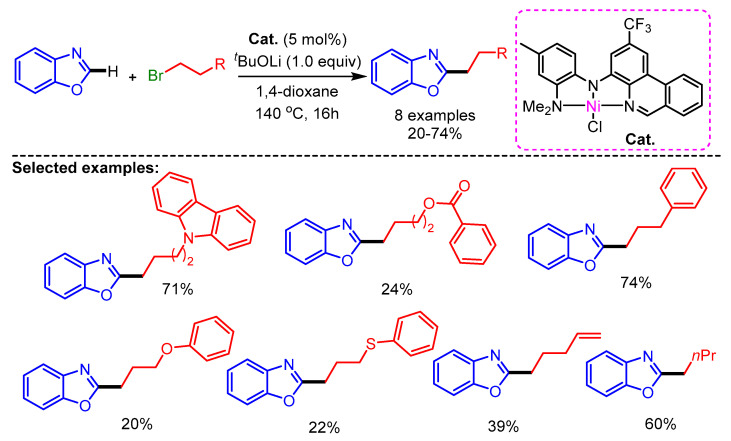
[Ni]-catalyzed alkylation of benzannulated azoles with alkylbromides.

Recently, Herbert and coworkers synthesized Ni(II)-complexes with phenanthridine-based ligands for the alkylation of azoles with alkyl halides [79]. In this work, a wide range of alkyl halides bearing different substituents such as carbazole, ester, aryl, arylether, thioether, alkenyl, and aliphatic groups show promising reactivity with benzoxazoles to generate the desired products in low to high yields (Scheme 47).

### 3.2. Secondary C–H Alkylation of Azoles

**Scheme 48:**
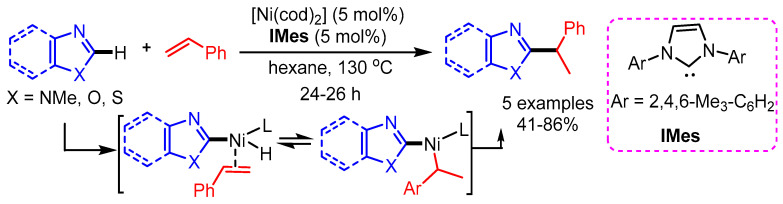
Ni/IMes-catalyzed hydroheteroarylation of azoles with styrene.

Although significant progress has been made for primary alkylation, the introduction of a secondary alkyl group is a much more difficult task. In 2010, Nakao et al. reported a Ni(0) catalyzed hydroheteroarylation of vinylarenes to give 1,1-diarylalkanes through oxidative addition of C–H bonds to heteroaryl groups [80]. [Ni(cod)_2_] and carbene ligand 1,3- dimesitylimidazol-2-ylidene (IMes) were employed as catalytic system in the nonpolar solvent hexane. A variety of vinylarenes that contain a phenyl group bearing both electron-rich and electron-poor substituents, reacted successfully. Several kinds of azoles including benzimidazole, benzoxazole, oxazole, and benzothiazole were also well tolerated, affording 1,1-diarylalkanes with modest to good yields (Scheme 48).

**Scheme 49:**
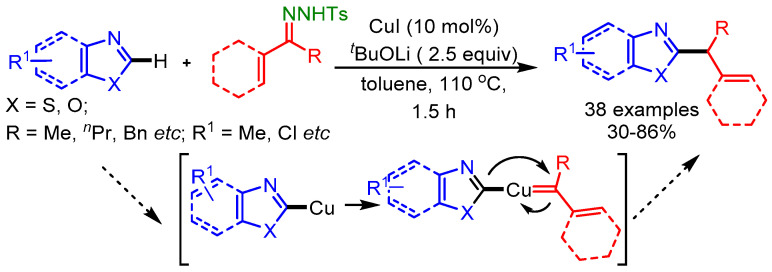
Cu-catalyzed direct benzylation or allylation of 1,3-azoles with *N*-tosylhydrazones.

In a pioneering work of Wang and coworkers, N-tosylhydrazones have been exploited as a secondary alkyl source for the direct alkylation of azoles with a Cu-catalyst, offering a general method to introduce a secondary alkyl group on an azole framework [81]. Under basic conditions, the authors proposed that a heterocycle-copper species was the intermediate, and a migratory insertion of the Cu carbene species was the key step in this Cu-catalyzed C–H functionalization reaction as shown in Scheme 49.

**Scheme 50:**
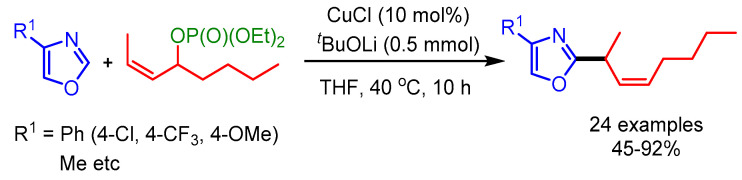
Cu-catalyzed allylic alkylation of electron-deficient heteroarenes with internal secondary allylic phosphates.

In 2012, Sawamura and coworkers extended this concept for the allylic alkylation of electron-deficient heteroarenes with internal secondary allylic phosphates resulting in excellent γ-regioselectivity and *E*-stereoselectivity (Scheme 50) [82]. In this reaction, steric factors played an important role in determining the regioselectivity of the product.

**Scheme 51:**
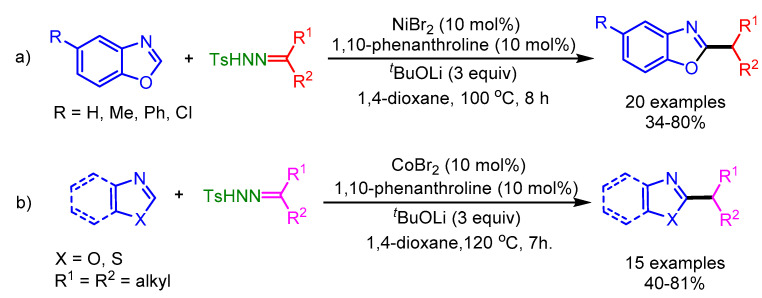
Ni- and Co-catalyzed direct alkylation of azoles with N-tosylhydrazones bearing unactivated secondary alkyl groups. (**a**) Ni-catalyzed; (**b**) Co-catalyzed.

Miura and coworkers also utilized N-tosylhydrazones as a radical source in a Ni-catalyzed alkylation of azoles [83]. The authors employed two different catalytic systems depending on the azole, i.e., a nickel catalyst for benzoxazoles and a Co(II) catalyst for 5-aryloxazoles and benzothiazoles (Scheme 51). Interestingly, the authors ruled out a carbene insertion pathway for this catalytic system. 

**Scheme 52:**
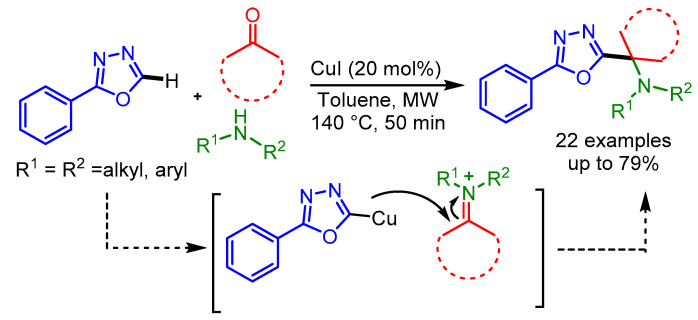
Cu-catalyzed direct secondary C–H alkylation of azoles.

In 2013, Van der Eycken and coworkers discovered a novel heteroarene-amine-ketone coupling (HAK-coupling) for the direct secondary alkylation of azoles [84]. This unprecedented Cu-catalyzed HAK coupling was operationally simple and allowed the facile installation of nitrogen-containing alkyl or alkaloid side chains on the azole moiety, using readily available starting materials. The HAK coupling reaction plausibly proceeded through the initial condensation of the aldehyde/ketone with the amine, leading to the corresponding iminium ion which was attacked by the azole–Cu to afford the final product (Scheme 52).

**Scheme 53:**
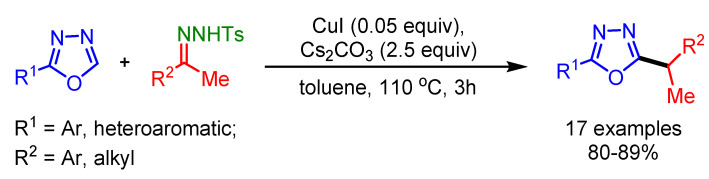
Cu-catalyzed direct cross-coupling of 1,3,4-oxadiazoles with *N*-tosylhydrazones.

Similar to Wang’s group report, in 2013, Das and coworkers also employed N-tosylhydrazones for the direct benzylation of aryl substituted 1,3,4-oxadiazoles in the presence of a Cu-catalyst [85]. A wide range of substituted oxadiazoles were prepared in high yields (Scheme 53). 

**Scheme 54:**
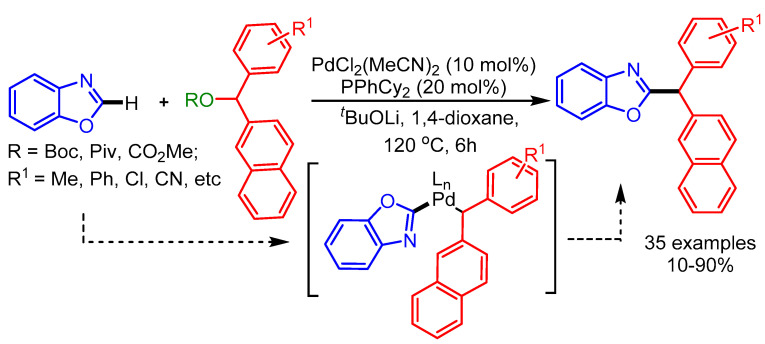
Pd-catalyzed C−H/C−O coupling of oxazoles and diarylmethanol derivatives.

In 2014, Miura and coworkers employed diarylmethyl carbonates or pivalates as coupling partners for the alkylation of oxazoles with a PdCl_2_(MeCN)_2_/PPhCy_2_ catalytic system [86]. This gives the access to challenging heteroarene-containing triarylmethanes (Scheme 54).

**Scheme 55:**
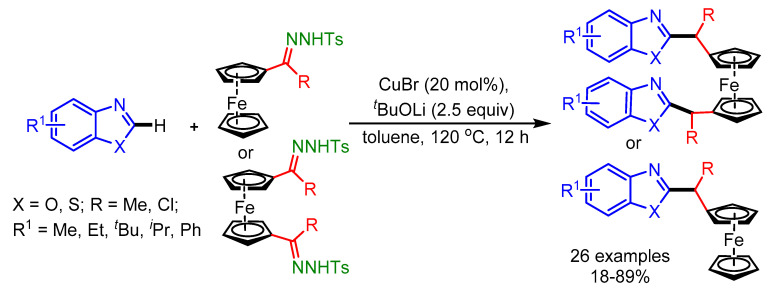
Cu-catalyzed cross-coupling of ferrocenyl ketone-derived *N*-tosylhydrazones with benzoxazoles.

In the same year, Chen and coworkers further evolved the direct C–H bond alkylation of azoles with ferrocenyl ketone-derived N-tosylhydrazones using an inexpensive CuBr catalyst [87]. Without using CuBr, the product was obtained in low yield, whereas replacement of CuBr with other transition metal salts slowed down the reaction. This catalyst system displayed good tolerance towards a range of functional groups on the ferrocenyl ketone derived *N*-tosylhydrazones (Scheme 55).

**Scheme 56:**
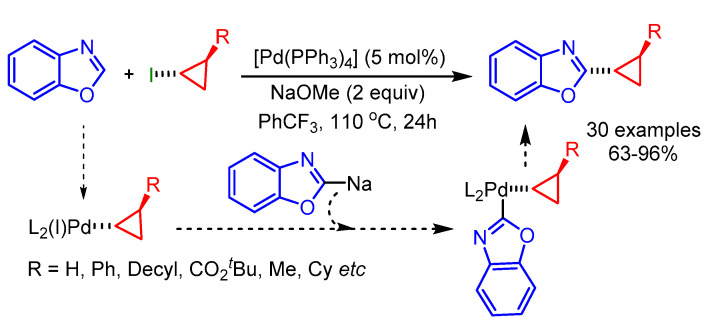
Pd catalyzed cyclopropylation of benzoxazoles with cyclopropyl halides.

In 2015, Zhou and coworkers reported a challenging cyclopropylation of benzoxazoles using stereoretentive cyclopropyl halides and a Pd(0) catalystic system [88]. The reaction started with the oxidative addition to the cyclopropyl C–X bond followed by transmetalation of an anionic heterocycle. Finally, reductive elimination delivered the desired coupling product (Scheme 56).

**Scheme 57:**
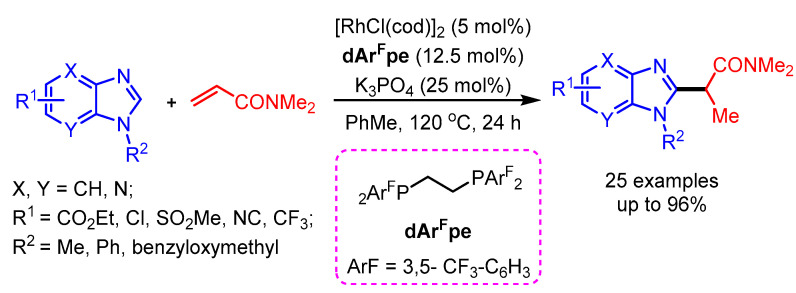
Rh(I)-catalyzed branched alkylation of benzimidazoles.

The first rhodium-catalyzed selective alkylation of benzimidazoles with *N*,*N*-dimethyl acrylamide was successfully developed by Ellman and coworkers in 2017 [89]. The combination of the electron-poor ligand dAr^F^pe with a Rh(I) catalyst in the presence of K_3_PO_4_ selectively delivered the alkylation product in high yields (Scheme 57).

**Scheme 58:**
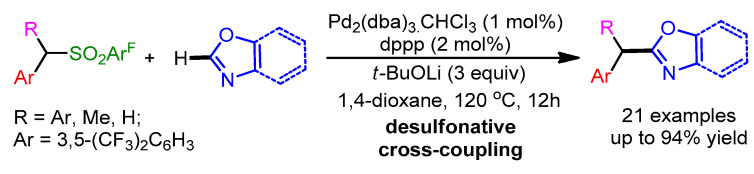
Pd-catalyzed desulfonative cross-coupling reaction of benzylic sulfone derivatives with 1,3-oxazoles.

In the same year, a Pd-catalyzed desulfonative deprotonative cross-coupling reaction of benzylic sulfone derivatives with 1,3-oxazoles was reported by Crudden and coworkers [90]. The presented methodology showed a high functional group tolerance and afforded excellent yields (Scheme 58).

**Scheme 59:**
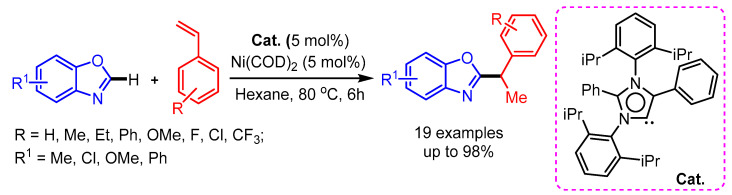
*N*-heterocyclic carbene and Ni(COD)_2_-catalyzed hydroheteroarylation of vinylarenes with benzoxazole.

In 2017, Mandal and coworkers developed a Ni(COD)_2_ catalyzed regioselective hydroheteroarylation of vinylarenes with benzoxazoles, providing an exclusively wide range of 1,1-diarylethane products [91]. The authors were able to isolate and characterize the active catalyst by single-crystal X-ray crystallography (Scheme 59).

**Scheme 60:**
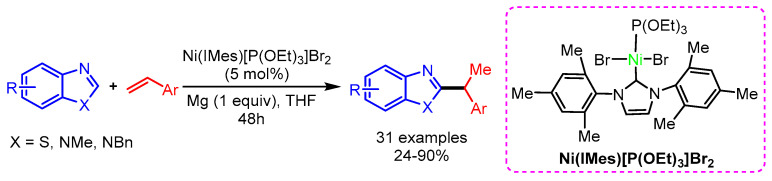
Ni-catalyzed hydroarylation of vinylarenes with benzothiazoles.

Similarly, Sun and coworkers also employed a NHCs-based nickel catalytic system for the regioselective hydroarylation of vinylarenes with benzothiazoles, affording the desired product in high yield [92]. In this reaction, the regioselectivity is controlled by the sterically demanding Ni(IMes)[P(OEt)_3_]Br_2_ catalyst (Scheme 60).

**Scheme 61:**
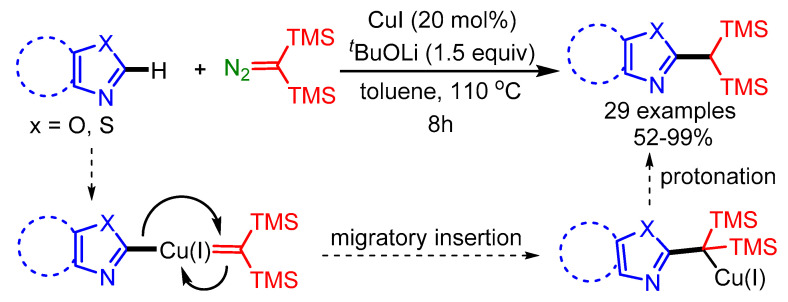
Cu(I)-catalyzed reaction of bis(trimethylsilyl)diazomethane with benzoxazoles and oxazoles.

In 2019, Wang and coworkers exploited an economical Cu(I)-catalyst to achieve the cross-coupling of bis(trimethylsilyl)diazomethane and benzoxazoles/oxazoles, affording a series of 1,1-bis(trimethylsilyl)-methylated heteroaromatic compounds in moderate to good yields, through a carbene migratory insertion process [93]. Mechanistic studies indicated that the main step is the formation of a Cu(I) carbene species, followed by migratory insertion and protonation to produce the final products, as shown in Scheme 61.

### 3.3. Tertiary C–H Alkylation of Azoles

**Scheme 62:**
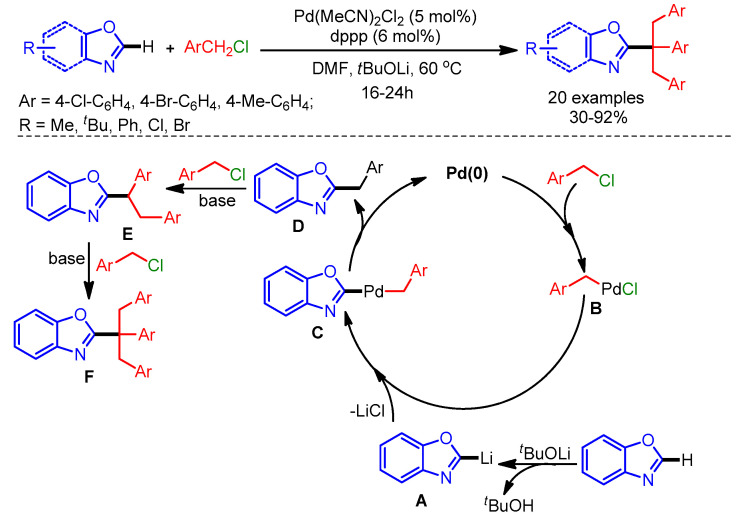
Palladium-catalyzed functionalization of azoles with a quaternary carbon center.

The inclusion of a tertiary alkyl group in the azole scaffold is challenging due to the steric effects and possible isomerization. In 2012, Xia and coworkers successfully reported a well-known Pd-catalyzed C–H benzylation of azoles employing benzyl chlorides to generate a quaternary carbon center [94]. Mechanistically, a Pd(0) species undergoes oxidative addition to generate the benzylpalladium(II) complex **B**, followed by a transmetalation step with lithium/azole species **A** to form intermediate **C**. Finally, reductive elimination delivers the mono-benzylated product **D**. The authors used Na_2_CO_3_ as base to synthesize the tribenzylated products via nucleophilic substitution with benzyl chloride (Scheme 62).

## 4. Alkynylation

**Scheme 63:**
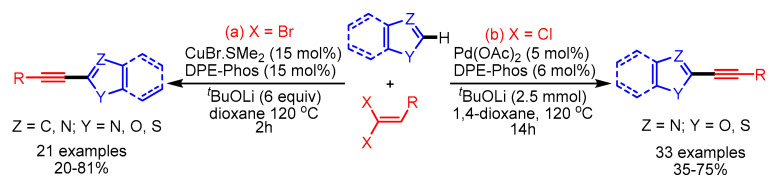
Transition metal-catalyzed direct alkynylation of azoles with 1,1-dihalo-1-alkenes.

The transition metal-catalyzed alkynylation of azoles provides a straightforward method to access diversely substituted azole acetylenes. In 2010, Piguel and coworkers disclosed copper-catalyzed direct alkynylation of various azoles with 1,1-dibromo-1-alkenes (Scheme 63, left) [95]. The authors proposed that the Cu(III)-intermediate readily undergoes reductive elimination to deliver the desired product in moderate to good yields under mild reaction conditions. Later in 2012, Zhu and coworkers reported alkynylations of oxazoles and benzothiazoles using a palladium catalytic system employing inexpensive *gem*-dichloroalkenes as user-friendly electrophiles with a broad scope (Scheme 63, right) [96].

**Scheme 64:**
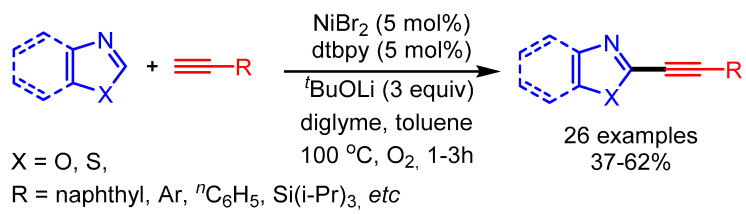
Ni-catalyzed direct alkynylation of azoles with terminal alkynes.

In 2010, Miura and coworkers reported a pioneering example of the metal-catalyzed direct C–H alkynylation of azoles with terminal alkynes. The reaction proceeded by using a stoichiometric or sub-stoichiometric quantity of copper. Subsequently, the same group further developed nickel-catalyzed direct alkynylation of azoles [97]. In the presence of a NiBr_2_·diglyme catalytic system with O_2_ as oxidant, they coupled various arylacetylenes with benzoxazoles to afford the desired products in low to moderate yields. In the proposed reaction mechanism, an (alkynyl)nickel intermediate, generated with Ni and a base, undergoes transmetalation with the heteroaryllithium. The desired product results after reductive elimination (Scheme 64).

**Scheme 65:**
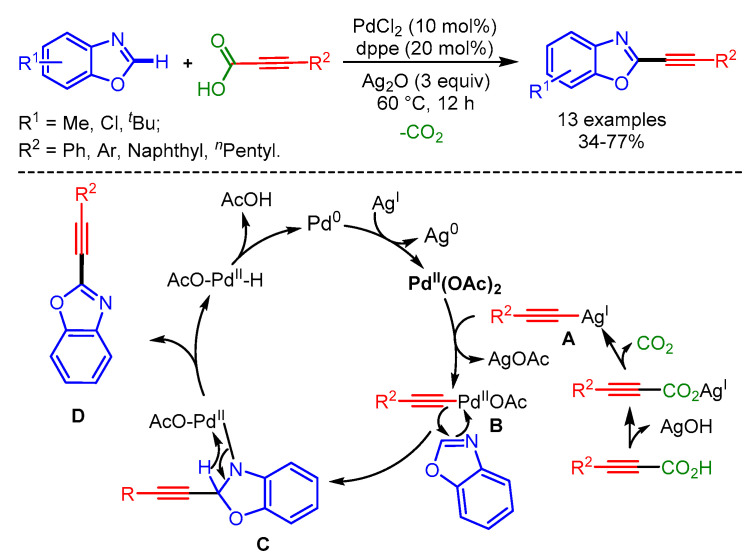
Pd-catalyzed decarboxylative C–H alkynylation of benzoxazoles with *α,β*-ynoic acids.

In 2013, Lee and coworkers exploited the well-known Pd catalytic system for decarboxylative C–H alkynylation of benzoxazoles with *α*,*β*-ynoic acids [98]. The authors showed the superior activity of 1,3-bis(diphenylphosphanyl)propane (dppp) as ligand for Pd to inhibit dimerization. The proposed mechanism involved (i) silver-oxide-catalyzed *α,β*-ynoic acid decarboxylation, (ii) transmetalation to form intermediate **B**, (iii) carbopalladation with the C–N double bond in the benzoxazole to afford **C**, and (iv) *β*-hydride elimination (Scheme 65).

**Scheme 66:**
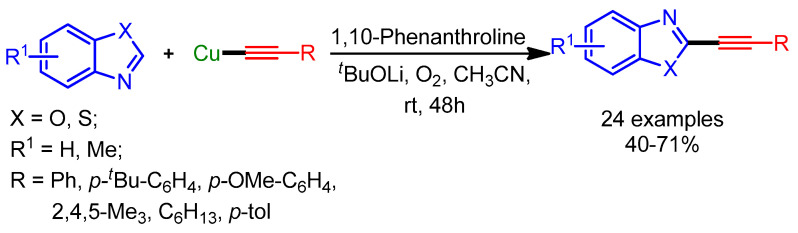
Direct alkynylation of arenes with Cu acetylides.

In 2014, Theunissen, Evano and coworkers developed an alternative strategy for the alkynylation of azoles with copper acetylides [99]. The use of preformed copper acetylides provided many advantages over other approaches, such as better functional group tolerance, mild reaction conditions, and the possibility of forming complex scaffolds (Scheme 66).

**Scheme 67:**
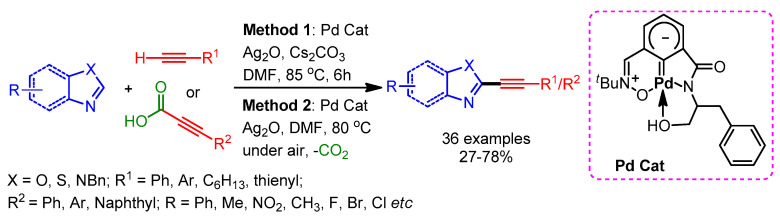
Dehydrogenative and decarboxylative C–H alkynylation of heteroarenes, catalyzed by a Pd(II)–carbene complex.

In 2015, Mannepalli and coworkers developed an air- and moisture-stable Pd(II) carbene complex for the alkynylation of azoles with terminal alkynes and aryl propiolic acids by cross-dehydrogenative or decarboxylative coupling, respectively [100]. A wide range of azoles including benzoxazoles, benzothiazoles, imidazoles, and benzimizoles afforded the desired alkynylation products in moderate to high yields (Scheme 67).

**Scheme 68:**
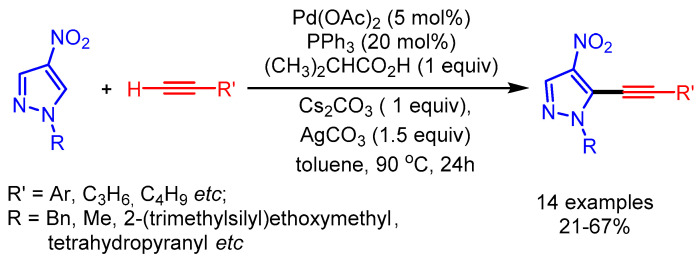
Palladium-catalyzed cross-coupling of nitropyrazoles with terminal alkynes.

Subsequently in 2018, Joo and coworkers further extended the substrate scope of the transition metal-catalyzed alkynylation of azoles with terminal alkynes [101]. The nitro group at the four-position of pyrazoles facilitates C–H cleavage to afford the desired alkynyl pyrazoles in moderate yields (Scheme 68).

**Scheme 69:**
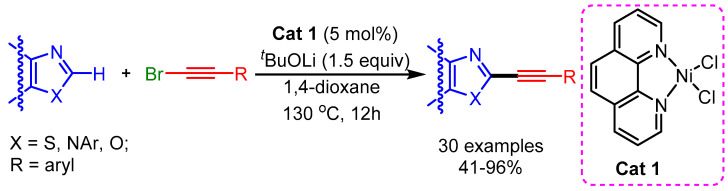
**N**i (II)-catalyzed C–H bond alkynylation of benzothiazoles and related azoles.

Recently, Punji and coworkers reported a Ni(II)-catalyzed C(2)–H bond alkynylation of (benzo)thiazoles, (benz)imidazoles, and oxazoles with alkynyl bromides, with the use of neither a copper cocatalyst nor phosphine ligands [102]. The reactions featured good functional group tolerance and wide substrate scope, despite the high temperature required for the reaction (Scheme 69).

## 5. Conclusions and Perspectives

In this review, we summarized recent developments in the transition metal-catalyzed direct C−H functionalization of azoles. These functionalizations are some of the most efficient methods to introduce various substituted olefins, alkanes, and alkynes to azoles. In addition to the importance of Pd in this field, researchers have widely explored several other transition metals such as Cu, Rh, Co, Ni, and Ru. The major advantage of transition metal-catalyzed direct C–H activation is that it requires no prefunctionalization of the starting azoles, resulting in, e.g., a wide scope, modularity, and high yields. Despite considerable advances, many challenges remain, e.g., overcoming the need for strong and harsh bases such as LiO and *^t^*Bu anions, and the unavoidable use of activated derivatives such as halo groups (Cl, Br, and I), carbonyl groups, and metal acetylides. Thus, compared with C−X/C−M coupling reactions, organic chemists should prefer catalytic activation of inert C–H bonds for new C–C bond formation reactions, starting from inexpensive and structurally diverse alkanes, alkenes, or alkynes. Furthermore, another challenge is to develop recyclable and highly efficient transition metal catalyst systems with low catalyst loading. Finally, researchers have partially solved the chemoselectivity and regioselectivity issues in these reactions by means of incorporating a directing group or using the sterics/electronics of the substrates. However, it is feasible to develop a suitable catalyst or fine-tune reaction conditions to change/control the regioselectivity.
